# Non‐apoptotic regulated cell death mediates reprogramming of the tumour immune microenvironment by macrophages

**DOI:** 10.1111/jcmm.18348

**Published:** 2024-04-23

**Authors:** Chengpeng Sun, Jianhao Zhan, Yao Li, Chulin Zhou, Shuo Huang, Xingen Zhu, Kai Huang

**Affiliations:** ^1^ Department of Neurosurgery The Second Affiliated Hospital, Jiangxi Medical College, Nanchang University Nanchang Jiangxi P. R. China; ^2^ HuanKui Academy, Jiangxi Medical College, Nanchang University Nanchang Jiangxi China; ^3^ The First Clinical Medical College, Jiangxi Medical College, Nanchang University Nanchang Jiangxi China; ^4^ The Second Clinical Medical College, Jiangxi Medical College, Nanchang University Nanchang Jiangxi China; ^5^ Institute of Neuroscience, Jiangxi Medical College, Nanchang University Nanchang Jiangxi P. R. China; ^6^ Jiangxi Key Laboratory of Neurological Tumors and Cerebrovascular Diseases Nanchang China; ^7^ JXHC Key Laboratory of Neurological Medicine Nanchang Jiangxi P. R. China

**Keywords:** autophagy, ferroptosis, immunotherapy, necroptosis, non‐apoptotic regulated cell death, pyroptosis, tumour immune microenvironment, tumour‐associated macrophages

## Abstract

Tumour immune microenvironment (TIME) plays an indispensable role in tumour progression, and tumour‐associated macrophages (TAMs) are the most abundant immune cells in TIME. Non‐apoptotic regulated cell death (RCD) can avoid the influence of tumour apoptosis resistance on anti‐tumour immune response. Specifically, autophagy, ferroptosis, pyroptosis and necroptosis mediate the crosstalk between TAMs and tumour cells in TIME, thus reprogram TIME and affect the progress of tumour. In addition, although some achievements have been made in immune checkpoint inhibitors (ICIs), there is still defect that ICIs are only effective for some people because non‐apoptotic RCD can bypass the apoptosis resistance of tumour. As a result, ICIs combined with targeting non‐apoptotic RCD may be a promising solution. In this paper, the basic molecular mechanism of non‐apoptotic RCD, the way in which non‐apoptotic RCD mediates crosstalk between TAMs and tumour cells to reprogram TIME, and the latest research progress in targeting non‐apoptotic RCD and ICIs are reviewed.

## 1 INTRODUCTION

Cellular morphological features, cellular surroundings and factors initiating cell death can be used as factors for cell death categorization. Cell death is mainly classified into accidental cell death (ACD) and regulatory cell death (RCD). Cell death is mainly classified into accidental cell death (ACD) and regulatory cell death (RCD) on the basis of cellular morphological features, cellular surroundings and factors initiating cell death, which are the main factors for cell death categorization. ACD is an uncontrolled cell death that occurs when a cell is subjected to a strong external stimulus, and RCD is a controllable cell death, the initiation of which is regulated by intracellular genes or molecules.^1^ RCD can be classified into apoptotic and non‐apoptotic types; the latter involves autophagy, ferroptosis, pyroptosis, necroptosis, parthanatos, entosis, cuproptosis and NETosis.^2^ Non‐apoptotic RCD has an important role in the effect of immune response, for example, inflammatory vesicles, a key component in pyroptosis, play a key role in glioblastoma (GBM) radiosensitization.^3^ Therefore, direct targeting of non‐apoptotic RCD in tumour cells has attracted more and more attention. Meanwhile, immune checkpoint inhibitors (ICIs), one of the immunotherapies, are limited by the immunosuppressive microenvironment and the reduced autoimmunogenicity of tumour cells cannot exert good efficacy, so the combination of ICIs and targeting non‐apoptotic RCD may be the solution for the current dilemma.^4^

The tumour microenvironment (TME) is an ecosystem where tumour cells, immune cells, tumour‐associated fibroblasts (CAFs) and endothelial cells are embedded in an extracellular matrix with plasticity. Notably, among the immune cell populations in the TME, macrophages are the most abundant class of immune cells, often referred to as tumour‐associated macrophages (TAMs).^5^ Numerous studies have demonstrated that TAMs play an important role in tumour growth, metastasis, immune escape and chemoresistance. Stimulated by TME, macrophages can change polarization selection between the M1 phenotype and the M2 phenotype. M1 macrophages can secrete pro‐inflammatory factors to induce anti‐tumour immunogenesis and inhibit tumour progression. Conversely, M2 macrophages secrete anti‐inflammatory factors that establish an immunosuppressive microenvironment and promote tumour progression.^6^, ^7^ Interestingly, most macrophages multipolarized to the M2 phenotype in large amounts of solid tumours. Therefore, a special focus on the specific pathways of macrophages in the regulation of the tumour immune microenvironment is of particular significance for targeted tumour therapy.

Tumour cell‐autonomous non‐apoptotic RCD can mobilize immune cells in the TME to improve the tumour immune microenvironment (TIME) and regulate tumour growth.^8^ Notably, the tumour cell death process with elicited adaptive immune responses in the TME is called immunogenic cell death (ICD).^9^ Tumour cells and TAMs, respectively, serve as the major cell population in the TME and the most abundant cells of immune cells in the TME. In addition, non‐apoptotic RCD mediates the crosstalk communication between TAMs and tumour cells at the cellular level, which is important for the shaping of TIME.^10^ Non‐apoptotic RCD regulates cellular communication between tumour cells and TAMs to reprogram TIME mainly via damage‐associated molecular patterns (DAMPs), cytokine secretion and the release of chemokine.^11^ DAMPs, which mainly consist of High Mobility Motif Box 1 (HMGB1), mitochondrial DNA and ATP‐binding immune cells or tumour cell surface receptors, have a dual function in tumour immunity.^12^

Therefore, here we reviewed autophagy, ferroptosis, pyroptosis and necroptosis mediate the horizontal crosstalk between tumour cells and macrophages in TME to reprogram the tumour immune microenvironment. In addition, we also reviewed the latest direct targeting of non‐apoptotic RCD and the latest pharmacological advances in the combination of direct targeting of non‐apoptotic RCD and ICIs.

## 2 OVERVIEW OF NON‐APOPTOTIC RCD

### 2.1 Overview of autophagy

Autophagy is a process in eukaryotic cells that regulates metabolic homeostasis in vivo, which is mediated by autophagy‐related genes (ATGs). Autophagy is divided into three categories: microautophagy, macroautophagy and molecular chaperone‐mediated autophagy (MCA). In a state of stress, cells will form autophagic membrane structures that engulf and degrade intracellular structures, including damaged organelles, unfolded proteins and pathogens, followed by fusion with lysosomes, and return of degraded products to the cytoplasm to maintain mass balance in the body.^13^

Autophagy is regulated by two ‘managers’, mTOR complex 1 (mTORC1) and AMP‐dependent protein kinase (AMPK).^14^ The mTORC1 can induce autophagy in response to nutrient deficiencies, oxygen deprivation and other unfavourable conditions in the body. In addition, during an energy deficit, cells activate AMPK to induce autophagy by sensing an increase in the ratio of AMP to ATP. When cells are under stress, mTORC1 and AMPK activate the ULK complex, which is mainly composed of FIP200, ATG13, ATG101, ULK1 and ULK2.^15^ The responsive element of the ULK complex is the Vacuolar Protein Sorting 34 (VPS34) complex. The combination of phosphatidylinositol 3‐phosphate kinase (PI3K) and the VPS34 complex generates phosphatidylinositol 3‐phosphate (PI3P), which is a marker that attracts PI3P‐binding molecules and plays a role in the LC3 lipidation and autophagosome formation.^16^ During the lipidation of LC3, ATG7, ATG3 and ATG5‐ATG12‐ATG16L are recruited.^17^, ^18^ Receptors bind to recruited proteins via ubiquitin labelling, which is the core of selective recruitment during autophagy. Subsequently, mediated by ATG9, the autophagosome becomes more complete, forming a separate space.^19^ Eventually, it is transported to the region near the lysosome, where it combines with the lysosome to form an autophagic lysosome to complete the degradation of the internal material. It is worth noting that the degraded material produces some nutrients that can be recycled.

There is a consensus that autophagy is a double‐edged sword. In the early stages of tumorigenesis, autophagy can eliminate cells with replication problems in a timely manner. However, after the tumour formation, the increased autophagic flux can help the tumour to adapt to the harsh environment such as nutrient deficiency and play a pro‐tumorigenic role. The effect of autophagy on tumours is complex, with both growth‐inhibitory and growth‐promoting properties. Initially, autophagy was thought to inhibit tumour growth for the following reasons. First, it was found that the awakening of proto‐oncogenes (PIK3CA) or the inactivation of oncogenes (PTEN) would inhibit the tendency of autophagy.^20^ Second, deletion of autophagy‐associated genes directs normal tissue towards cancer. For example, the deficiency of ATG5 or ATG7 in mice results in benign hepatocellular carcinoma, suggesting that specific autophagy defects will promote tumorigenesis.^21^ The opposite view is that autophagy promotes tumour growth, and the earliest evidence for this comes from the finding that in hypoxic and nutrient‐poor TME; autophagy is upregulated to allow tumour cells to better survive against apoptosis and necrosis.^22^ Importantly, autophagy plays a pro‐ or anticancer role with environment‐dependent (tumour microenvironment and immune microenvironment) characteristics. The functional characteristics of autophagy differ in different types of tumours, different gene mutations and even different research models.^23^

### 2.2 Overview of ferroptosis

The concept of ferroptosis was introduced in 2012 as a form of iron‐dependent cell death due to lipid peroxidation. This cell death has two main core programs: lipid peroxidation and iron accumulation. The cellular morphology of ferroptosis is characterized by loss of cell membrane function, swelling of cytoplasm and organelles, proper condensation of chromosomes, condensation and decreased number of mitochondria, decreased or absent cristae and increased density of outer membrane.^24^ In terms of chemical alterations, there is mainly an increase in ROS concentration, elimination of glutathione and decreased cysteine uptake and nicotinamide adenine dinucleotide phosphate (NADPH).^25^ Therefore, ferroptosis brings the cell to its end mainly through oxidative damage.

**FIGURE 1:**
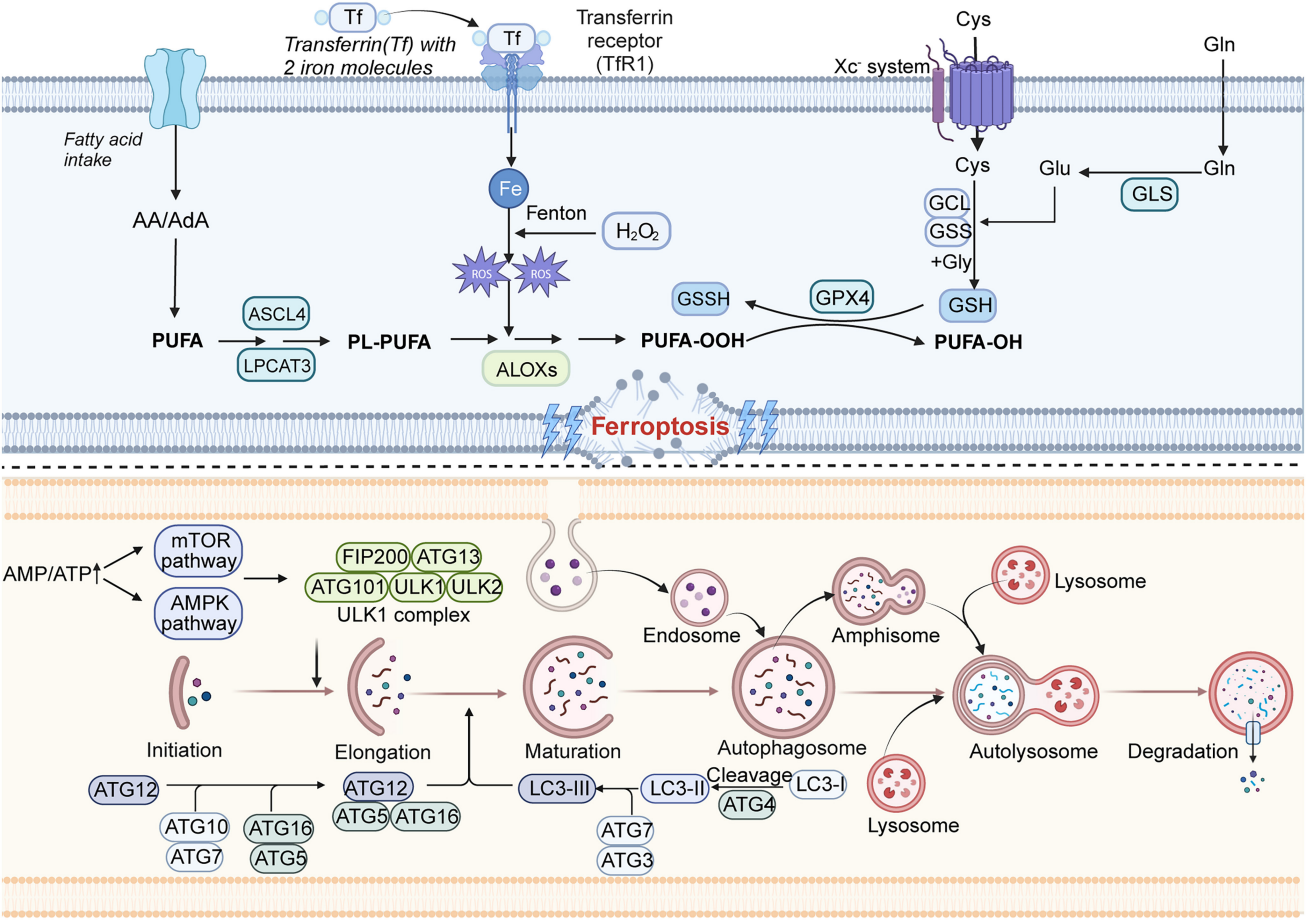
Mechanism of ferroptosis and autophagy. Above is the regulatory pathway of iron death. Intracellular iron overload leads to the accumulation of ROS, which in turn peroxides polyunsaturated fatty acids on the cell membrane and damages the cell membrane. The Xc‐system of cell membrane can transport cystine into the cell to synthesize GSH, and under the catalysis of GPX4, it can remove ROS from the cell and reduce the peroxidized polyunsaturated fatty acids. These two systems are antagonistic to each other and jointly regulate iron death. Below is the regulation pathway of autophagy. Cells sense ATP/AMP signals in the surrounding environment, activate downstream signal transduction through mTOR and AMPK related signal molecules and induce the formation of autophagy. Then autophagy combines with lysosomes to degrade the internal substances and release nutrients for cell recycling.

The mechanism of ferroptosis is mainly an increase in iron influx and a decrease in output while intracellular iron is released so the increased intracellular iron concentration induces iron metamorphosis.^26^ In more detail, transferrin (TF) binds to transferrin receptor (TFRC) and increases extracellular iron uptake, which, together with the autophagic degradation of intracellular iron storage proteins or iron export transporter proteins solute carrier family 40 member 1 (SLC40A1), increases iron accumulation.^27^ Interestingly, lactotransferrin 40 (LTF) also increased ferroptosis effects by increasing iron uptake.^28^ The accumulation of iron reacts with hydrogen peroxide via the Fenton reaction and leads to high production of ROS, which in turn activates lipoxygenases (ALOXs). Polyunsaturated fatty acids (PUFA) are first converted to phospholipid‐polyunsaturated fatty acids (PL‐PUFA) by long‐chain fatty acid‐CoA ligase 4 (ACSL4) and lysophospholipid acyltransferase 3 (LPCAT3) and then altered to phospholipid hydroperoxides (PLOOH) by ALOXs.^24^ The existence of oxidative systems in cells is important for antagonizing the complex signalling network of cytokines that undergo ferroptosis. The xc‐System‐glutathione (GSH)‐glutathione peroxidase 4 (GPX4) is a classical intracellular antioxidant pathway,^26^ which is regulated bybrca1‐associated protein 1 (BAP1) and P53.^29^ The xc‐system is an antioxidant protein composed of SLC7A11 and SLC3A2. GSH, as an irreplaceable ‘helper’ of GPX4, has an xc‐system (mainly responsible for intra‐ and extracellular transport of cystine and glutamate) dependent reduction of PLOOH to fatty alcohols, and such self‐help is important for cells.^30^ Interestingly, autophagy can induce the onset of ferroptosis. It has been reported that nuclear receptor coactivator 4 (NCOA4) can induce ferritin autophagy and RAB7A (a member of the RAS family) can significantly induce lipid autophagy.^31^, ^32^ In the presence of both, intracellular levels of iron and free fatty acids were significantly increased inducing ferroptosis. After further research on ferroptosis, ferroptosis inducers have been found to have significant effects in limiting tumour progression. For example, the well‐known ferroptosis inducer (erastin), which targets the xc‐system, has been shown to be highly effective in treating KRAS mutation‐induced pancreatic cancer^33^ (Figure 1).

### 2.3 Overview of pyroptosis

Pyroptosis, one of the non‐apoptotic RCDs, is a type of cellular immunogenic death associated with inflammatory responses. The main cytological features of pyroptosis are cell expansion, plasma membrane rupture, chromatin breaks and release of large amounts of intracellular pro‐inflammatory substances caused by caspase family‐dependent cleavage of the Gasdermin family.^34^

Intracellular pyroptosis has been reported to be achieved through both classical and nonclassical pathways. In the classical pathway, it is usually believed that pro‐caspase‐1 becomes an active state of caspase‐1 due to the activation of inflammatory vesicles. Inflammatory vesicles are complex multimeric complexes that help protect organisms from endogenous risk factors and pathogen attacks.^35^ Inflammatory vesicles are composed of three main structures: sensor proteins with pattern recognition receptor roles (PRRs), junction proteins (ASCs) and downstream caspase families.^36^ Different types of sensor proteins mediate the initiation of pyroptosis by receiving different exogenous or endogenous stimuli. For example, NLRP3 inflammatory vesicles commonly sense danger signals from within the cell to mediate pyroptosis. There are five main types of common inflammatory vesicles in the classical pathway of pyroptosis: NLRP3 inflammatory vesicles, AIM2 inflammatory vesicles, NLRP1 inflammatory vesicles, PYRIN inflammatory vesicles and NLRC4 inflammatory vesicles.^36^ Caspase‐1 cleaves the GSDMD proteins to form the active N‐terminal (GSDMD‐NT) and C‐terminal (GSDMD‐CT), while GSDMD‐NT also has a role in promoting IL‐1β and IL‐18 production.^37^ The active N‐terminus binds to acidic phospholipids in the plasma membrane to form tiny voids that alter the intracellular osmotic pressure and thus promote pyroptosis.^38^ Unlike the classical pathway, the nonclassical pathway does not require inflammatory vesicles to activate caspases, and the two pathways require different types of caspases. In the nonclassical pathway, cells can bind with LPS (lipopolysaccharide) via transmembrane TLR4 (Toll‐like receptor 4) and then stimulate pro‐caspase‐4, pro‐caspase‐5 and pro‐caspase‐11 to become active caspase‐4/5/11 and thus cleave GSDMD proteins to GSDMD‐NT and GSDMD‐CT.^38^ Interestingly, activated caspase‐11 induces the effect of rupture of the pannexin‐1 channel which leads to the release of ATP. Next, the release of ATP activates P2X7 channels and leads to the efflux of K+, which ultimately binds to Nima‐associated kinase 7 (NEK7) to activate the NLRP3 inflammasome and induce the secretion of IL‐1β.^39^, ^40^ In addition to the above forms, apoptosis‐associated caspases (caspase3/6/8) are also major players in pyroptosis. It has been reported that apoptosis‐related caspase‐3 and caspase‐8 can also cleave GSDMD proteins to initiate pyroptosis.^41^, ^42^ Notably, activation of TLR, IL‐1R and TNFR can contribute to the elevation of caspase‐8 expression, which in turn upregulates the expression of NLRP3 inflammasome and IL‐1β through the signalling pathway of NF‐κB.^43^ In addition, caspase‐6 regulates NLRP3/caspase‐1 activity to regulate pyroptosis through the interaction of RIPK3 in combination with ZBP1 as a signalling axis.^44^

It is well known that pyroptosis is a double‐edged sword in cancer, which can manifest a role either in promoting or inhibiting tumour development. Specifically, after pyroptosis, the cells promote the release of cytokines such as IL‐1β and IL‐18, which can have an effect on tumour development and metastasis.^45^ For example, in non‐small cell lung cancer (NSCLC), the pyroptosis inducer polyphyllin VI (PPVI), a major saponin isolated from Trillium tschonoskii Maxim (TTM), mediated the occurrence of caspase‐1‐initiated pyroptosis through the ROS/NF‐κB/NLRP3/GSDMD signalling axis and inhibited the proliferation of NSCLC.^46^ In contrast, in prostate cancer, IL‐1β can mediate the metastasis of prostate cancer by activating IL‐8 through the MAPK pathway.^47^ Altogether, these examples demonstrate the dual role of pyroptosis in tumour progression, which largely depends on the type of cancer, the immune status of the host and the relevant effector molecules.

### 2.4 Overview of necroptosis

Since necroptosis does not rely on caspase cleavage and apoptosis, it can also be regarded as a backup cell death pathway of apoptosis. Briefly, necroptosis mainly relies on the involvement of receptor‐interacting protein 1 (RIP1), RIP3 and mixed‐lineage kinase‐like domains (MLKL), in which inhibition of caspase‐8 proteins is critical for necroptosis.^48^ Necroptosis is initiated mainly by PRR (FasL, TNFR1, TLR3, TCR), which can sense stimuli from the intracellular and extracellular environment.^49^ Interestingly, the role of hypoxia in necroptosis cannot be ignored.^50^ TNFα/TNFR is the most classical and well‐studied pathway in necroptosis, and by introducing this molecular pathway, the mechanism of necroptosis can be understood. The process of necroptosis is divided into three main steps: complex I formation, complex II assembly and necroptosis vesicle assembly. First, upon the combination of TNFα and TNFR1, multiple proteins are recruited, including RIPK1, TRADD (TNFR‐associated death structural domain), cIAP1 (apoptosis inhibitory protein 1), cIAP2, TRAF2 (TNFR‐associated factor 2) and TRAF5, which are assembled into complex I.^51^ Notably, RIPK1 in complex I can be polyubiquitinated by cIAP1/2 to initiate the cellular NF‐κB pathway and induce cell survival.^52^ Next, complex I was modified to complex II in response to cylindromatosis (CYLD), consisting of RIPK1, TRADD, caspase‐8 and FADD (FAS‐associated death structural domain) protein.^53^ What is of critical importance is whether caspase‐8 is inhibited; when caspase‐8 remains active, cells do not proceed towards necroptosis. However, when caspase‐8 function is inhibited, RIPK1, RIPK3 and MLKL are phosphorylated and then sequentially linked to assemble necroptosis vesicles.^54^ MLKL eventually arrives at the plasma membrane and disrupts the plasma membrane structure, assisting the onset of necroptosis.

**FIGURE 2:**
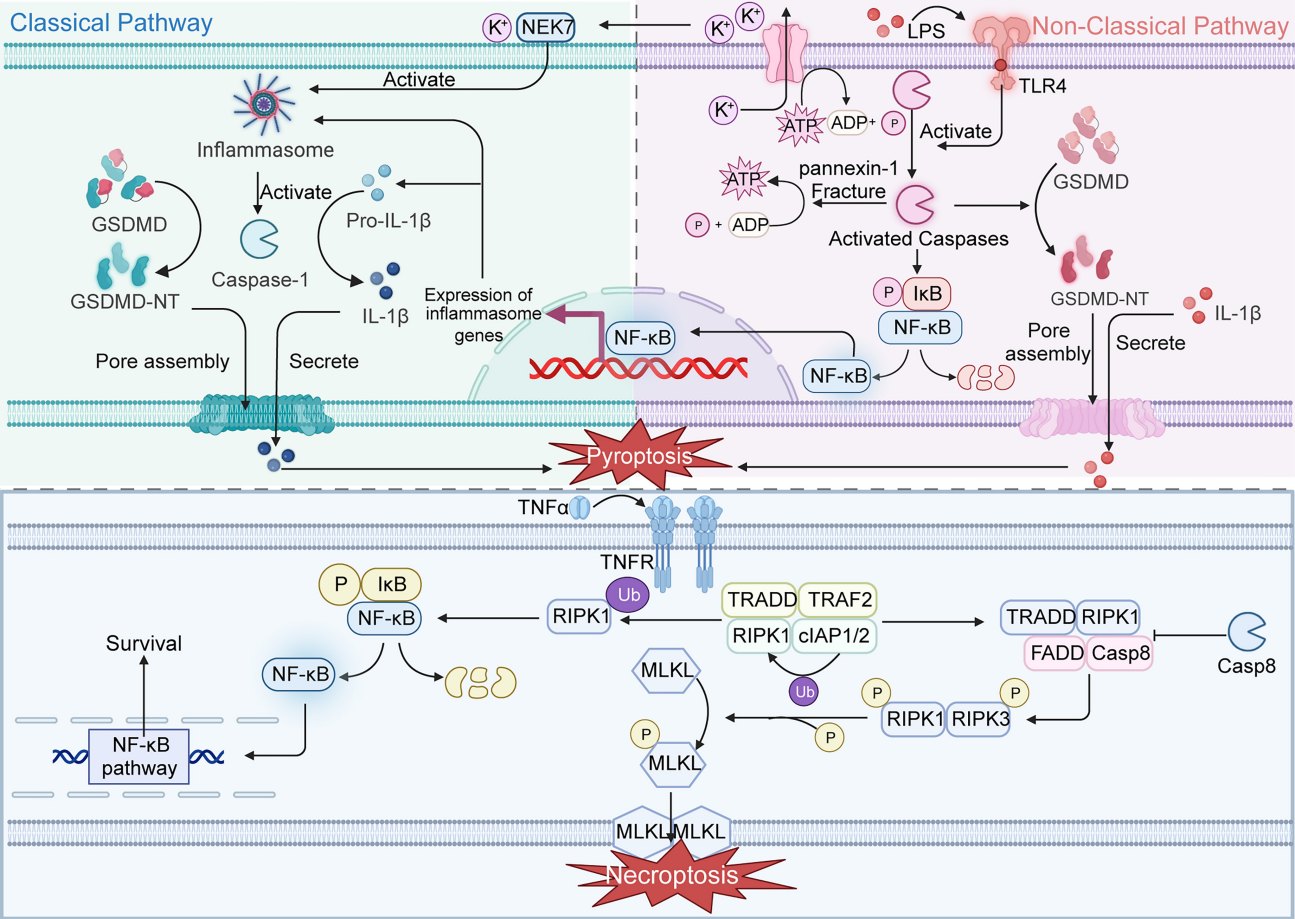
Mechanism of pyroptosis and necroptosis. Above is the regulation pathway of pyroptosis. The activation of inflammatory corpuscles will activate intracellular caspases. GSDMD protein is cleaved by activated caspases to form GSDMD‐NT with perforation activity, which forms tiny pores on the cell membrane, changes the osmotic pressure inside the cell and promotes the occurrence of apoptosis. At the same time, inflammatory factors activated in cells will also be released to the extracellular space through pores, triggering inflammatory reactions. Below is the regulatory pathway of necroptosis. After TNFR on the cell membrane binds to the ligand, it starts the assembly of intracellular complex and phosphorylates the downstream MLKL. Phosphorylated MLKL has perforation activity, forming pores on the cell membrane and inducing necrotic apoptosis.

Like autophagy and pyroptosis, necroptosis has two sides to tumour development, acting as both a partner and a competitor. The supporting rationale for being a competitor is the downregulation or absence of the expression of many necroptosis‐related key genes in tumours. Specifically, after exploration in more than 60 cancer cell lines, RIPK3 protein expression was found to be downregulated or absent.^55^ For example, RIPK3 knockout mice were reported to be more susceptible to colorectal cancer compared to normal mice, and more pro‐tumorigenic factors were found in the TME.^56^ Notably, the expression of necroptosis‐related genes is not always downregulated in tumours. For example, RIPK1 expression is significantly elevated in glioblastoma (GBM), which is also associated with poor prognosis of glioblastoma.^57^ However, as with ICD, necroptosis supports tumour progression by suppressing the immune microenvironment and promoting cancer metastasis. RIPK3 has been reported to modulate the secretion of cytokines in the TME and thus mediate the innate and adaptive immune systems to regulate immune homeostasis and promote tumour progression.^58^ In summary, the role of necroptosis in cancer may be related to the immune status of the host, the type of tissue, stress status and the disease environment, and is by no means a simple one‐size‐fits‐all issue. A better understanding of the role of necroptosis in cancer could help us find relevant therapeutic targets (Figure 2).

### 2.5 Overview of other non‐apoptotic regulated cell death

Parthanatos is characterized by a high degree of poly (ADP‐ribose) (PAR) dependence and estrangement from the caspase proteins family. The PAR family is capable of repairing damaged DNA. When DNA is heavily damaged, a dramatic increase in the activity of PAR (mainly PARP‐1) can mediate the development of parthanatos.^59^ The occurrence of parthanatos is divided into several steps, including PAR overactivation, PAR binding to apoptosis‐inducing factor (AIF), the release of AIF from mitochondria and AIF‐mediated chromatid cleavage. Notably, macrophage migration inhibitory factor (MIF) and AIF complex promote the release of AIF and mediate DNA cleavage, which is the second pathway of parthanatos occurrence.^59^

Entosis is a non‐phagocytic process, and unlike phagocytosis by macrophages, entosis is more proactive. In addition, entosis is characterized by the formation of cell‐in‐cell structures, with the prerequisites of cell adhesion and cytoskeletal rearrangement pathways (actin, RhoA and rock).^60^ Most entotic cells cannot escape the fate of being killed in phagocytes, which is called entotic cell death. The main modes of entotic cell death include lc3‐related phagocytosis (LAP) and lysosomal degradation mediated by cathepsin B (CTSB).^61^

Cuproptosis is an RCD that occurs when excessive accumulation of intracellular copper leads to a dysfunction of the mitochondrial respiratory regulatory system. More specifically, the high expression of SLC31A1 (input port) and the low expression of ATP7A/B (output port) usually lead to the increase of copper content in cells.^62^ Thiolated proteins are oligomerized after binding with copper, and the loss of a large number of Fe‐S cluster proteins leads to a proteotoxic stress response; thus, the cuproptosis is completed. In addition, GSH can bind copper and reduce intracellular copper content, thus potentially preventing cuproptosis.^62^

NETosis is a major mode of inflammatory cell death with NET as the initial factor. Nets are secreted primarily by activated neutrophils, which encapsulate pathogens and expose them to high concentrations of effector proteins. Besides neutrophils, other types of white blood cells (mast cells, eosinophils) and cancer cells have the characteristics of making NETs in response to external stress.^63^ Notably, the dynamic evolution of NETosis can be divided into several phases: ROS production, neutrophil elastase (NE), anti‐myeloperoxidase antibodies (MPO), histone modification and chromatin degradation.^64^

## 3 NON‐APOPTOTIC RCD MEDIATES TIME REPROGRAMMING OF MACROPHAGES

### 3.1 Autophagy mediates TIME reprogramming of macrophages

It is well known that insufficient oxygen supply due to excessive growth of solid tumours is very likely to lead to hypoxia inside the tumour, and thus, hypoxia is one of the common features of solid tumours. Tumour cells in an anoxic TME therefore trigger an autophagic effect that has an irreplaceable impact on the subsequent immune response. Recent studies have reported that autophagy can remodel TIME by modulating the release of DAMPs in dying or injured tumour cells and that the impact caused by DAMPs in the TME inversely enhances the intensity of autophagy in cancer cells. For example, a high concentration of ATP in the tumour extracellular environment can recruit more immune cells to construct TIME to participate in anti‐tumour immunity. Autophagy, as an ICD modality, can help tumour cells enhance ATP secretion by means of intracellular lysosomal transport.^65^ Therefore, for autophagy‐deficient tumours, a decrease in the number of recruited immune cells due to a decrease in extracellular ATP concentration can lead to a decrease in the effectiveness of anti‐tumour response.^66^ HMBG1, a classical DAMP, can conjugate with Toll‐like receptor 2 (TLR2) on the macrophage surface, leading to NADPH oxidase‐2 (NOX2)‐mediated autophagy pathway by generating ROS. This ultimately leads to inactivation of NF‐κB p50/p65 and increased P50/P50 homodimer expression, which can, respectively, promote macrophage differentiation towards M2 type and inhibit the production of pro‐inflammatory factors, leading to a suppressive development of TIME.^67^ It is noteworthy that the selection of an additional TLR4 receptor by HMBG1 activates mTOR to inhibit autophagy mediated by NF‐κB and avoids polarization of the M2 phenotype.^15^

Tumour cell‐associated autophagy can help them escape innate and adaptive immunity. It has been reported that autophagy in tumour cells can affect macrophage phagocytosis by increasing CD47 expression through autophagy.^68^ The high expression of CD47 can bind to SIRPα, which is on the surface of macrophages, inhibiting their antigen presentation and disrupting anti‐tumour immune responses. Furthermore, autophagosomes (TRAPs), an LC3‐II+ double‐membrane extracellular vesicle (ev) secreted by tumour cells, can promote macrophage differentiation towards the M2 phenotype, inhibit the infiltration of CD4+ and CD8+ T cells in the TME and get involved in the construction and maintenance of the immunosuppressive microenvironment.^69^ It has been demonstrated that silencing of the autophagy gene Beclin1 reduces the secretion of TRAPs and leads to a significant delay in tumorigenesis, which may be strongly correlated with the decreased capacity of reprogrammed macrophages. Low immunogenicity and reduced CTL infiltration may be the main pathways by which autophagy helps cancer cells escape adaptive immunity.^70^ Interferon type I (IFN‐I) plays a significant role in the recruitment of antigen‐presenting cells (APCs) and the promotion of maturation, and IFN gene‐stimulating factor (STING) has an irreplaceable role in the activation of IFN‐1.^71^, ^72^ However, the activity of STING is decreased in TME due to the inhibitory effect of SOX2, which blocks the secretion of IFN‐1. In addition, this inhibitory effect of SOX2 can be enhanced by the rise of autophagic flux in tumour cells.^73^ Therefore, inhibition of autophagy can significantly increase the level of IFN‐1, which will promote the activation of CXCL10 and ultimately increase CD8+ T‐cell infiltration in TME. One example is, tumour cells degrade MHC‐I molecules in pancreatic ductal carcinomas via the autophagy tool NBR1, aiding in tumour immune escape.^74^ In summary, autophagy in tumour cells is a powerful contributor to helping cancer cells escape innate and adaptive immunity. It is renowned that the binding of PD‐L1 on the surface of tumour cells and PD‐1 on the surface of T cells leads to the inactivation of T cells, thus achieving the goal of immune escape.^75^ Interestingly, more and more studies have shown us that autophagy in tumour cells can influence the efficiency of immune escape by degrading immune checkpoints.^76^ However, ‘smart’ tumours have derived their own countermeasures. By modifying the transcription of the autophagy gene, the decrease of immune escape efficiency caused by autophagy was inhibited; thus, the ability of immune escape was restored. For example, in a colorectal model, acyltransferase (DHHC3) affects PD‐L1 autophagy to maintain immune escape capacity while potentially promoting tumour development.^77^

Autophagy possesses a non‐negligible role in immune cell recruitment, macrophage antigen extraction capacity, direction of polarization and TME remodelling. First, autophagy has the ability to regulate the levels of MHC‐I and MHC‐II expression in macrophages and mediate the effect of macrophages on TME. Autophagy inhibits MHC‐I expression while promoting MHC‐II expression. The expression of MHC‐I was reported to be elevated in ATG5−/− and ATG7−/− macrophages, but the RNA content was the same compared to the autophagy intact gene. This demonstrates that autophagy promotes a pathway for lysosomal degradation of MHC‐I and leads to a decline in macrophage antigen presentation capacity.^78^ In contrast, oxidized LDL activated autophagy by mediating MAPK8/9 phosphorylation, which was found to result in elevated MHC‐II expression.^79^ Interestingly, although also as one of the APCs, the antigen‐presenting capacity and induction of anti‐tumour immunity of TAM were not superior compared to dendritic cells (DCs) and may even lead to abnormal proliferation of T cells.^80^, ^81^ Moreover, autophagy can modulate macrophage phenotype. In a hepatocellular carcinoma (HCC) model, the natural compound baicalin was reported to activate the autophagic pathway to adjust the polarized phenotype of macrophages to control cancer progression.^82^ LC3‐associated autophagy (LCA), as a pathway different from classical autophagy, is involved in the clearance of apoptotic cells by T‐cell immunoglobulin mucin 4 (TIM4), which acts as a receptor to affect macrophage polarization., The process that TIM4 is a receptor for macrophages has the ability to help clear apoptotic cells is called efferocytosis.^83^ Efferocytosis exhibits selective control of anti‐inflammatory and pro‐inflammatory cytokines, such as increasing the secretion of anti‐inflammatory cytokines IL‐10, TGF‐β, etc., while inhibiting the secretion of pro‐inflammatory cytokines IL‐6, and IL‐1β. Although this ‘unfair’ regulation mechanism is beneficial to macrophages to play a better role in phagocytosis and cleaning, it will also promote macrophages to polarize to M2 and create an inhibitory TIME.^84^ In TME, IL‐6 and CCL2 trigger autophagy, respectively, by binding to interleukin 6 receptor (IL‐6R) and CC chemokine receptor 2 (CCR2) and induce macrophage towards M2 type, which is strong support for maintaining inhibitory TIME and promoting tumour progression.^85^, ^86^ Collectively, TAM provides strong support for the development of immune tolerance in tumour progression and is enhanced by autophagy in TAM. Nevertheless, the specific local property of TME is more likely to trend macrophage differentiation towards the M2 type; however, in non‐progressive, degenerative and early tumours, it is also possible that some key autophagy regulatory genes are suppressed, which will lead to the polarization direction of macrophages developing towards M1 type.^87^, ^88^ These examples demonstrate that macrophage polarization is inextricably linked to local TME.

**FIGURE 3:**
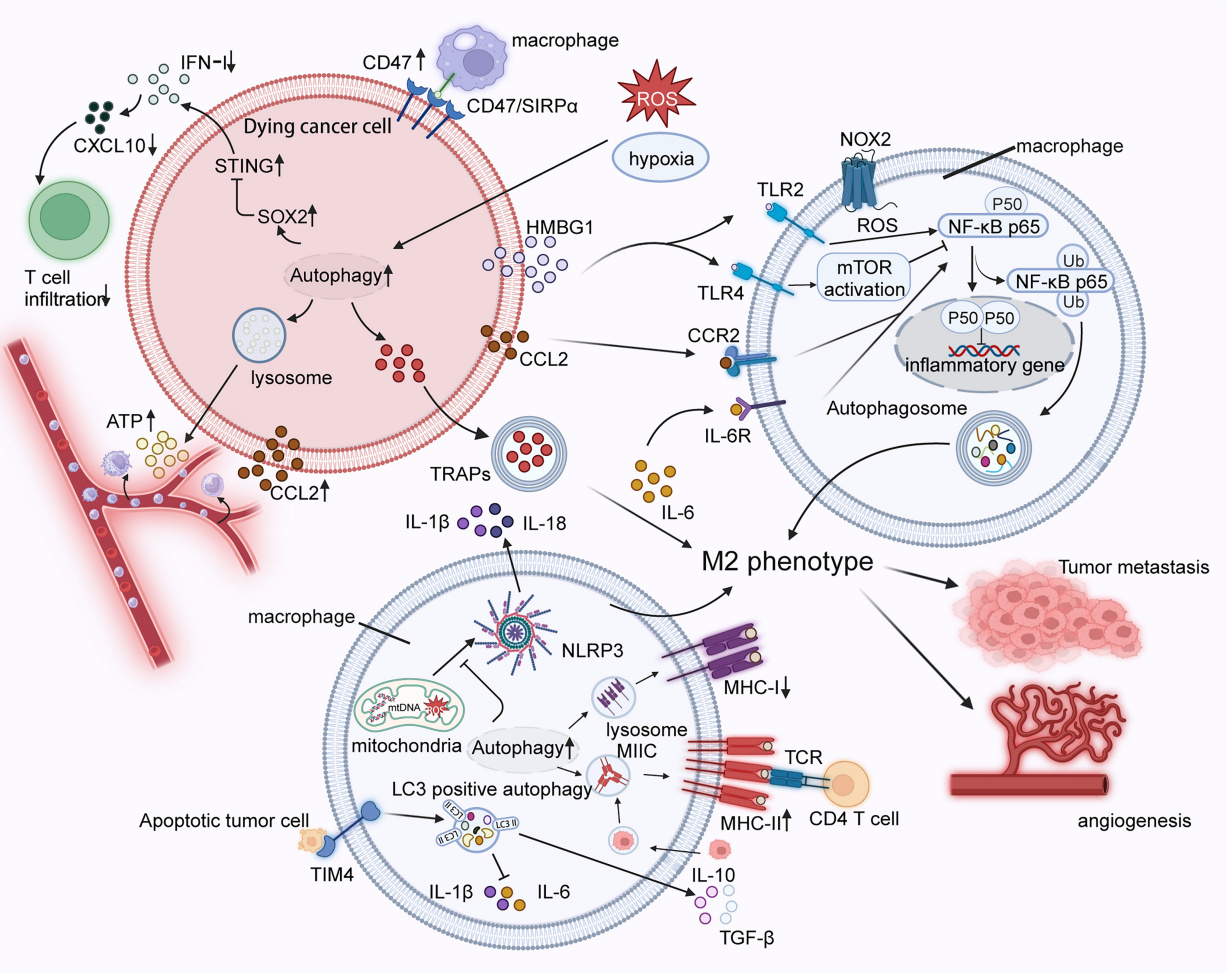
Autophagy mediates macrophage reprogramming of the tumour immune microenvironment hypoxia and ROS in TME initiate macrophage autophagy, increase extracellular ATP concentration, promote NK cell and macrophage infiltration and reduce IFN‐1 secretion and T‐cell infiltration. At the same time, the phagocytosis of macrophages was reduced by CD47/SIRPα. DAMPs, CCL2 and IL‐6 can also trigger macrophage NF‐κB to mediate autophagy, chemotactic macrophage differentiation to M2 phenotype and reduce the release of inflammatory factors. Notably, autophagy‐secreted TRAPs also can chemotactic the M2 phenotype of macrophages. In addition, the enhancement of macrophage autophagy can effectively block the accumulation of DAMPs in mitochondria and avoid starting the focal death pathway to secrete pro‐inflammatory factors such as IL‐1β and IL‐18. The clearance of apoptotic cells by TIM4 will trigger autophagy mediated by LCA pathway in macrophages to promote the secretion of anti‐inflammatory factors and inhibit the secretion of pro‐inflammatory factors. To sum up, the effect of autophagy secretion of tumour cells on macrophages and autophagy of macrophages themselves will promote the polarization of macrophages into M2 phenotype and promote tumour metastasis and angiogenesis.

Recent studies have reported that autophagy regulates the formation of inflammatory vesicles, essential progress that is required for the formation of pro‐inflammatory factors IL‐1β and IL‐18, to mediate the release of inflammatory mediators.^89^ Based on this, selective autophagy in mitochondria, an instinctive response against stressful stimuli, can help to clean up damaged mitochondria and avoid the accumulation of mitochondria‐derived DAMPs (ROS and mtDNA), which can activate the inflammasome vesicles to promote the release of pro‐inflammatory mediators such as IL‐1β and IL‐18.^90^ In fact, IL‐1β promotes glycolysis in gliomas through the IL‐1β‐protein kinase‐δ (PKCδ)‐glycolysis enzyme‐3‐phosphate dehydrogenase (GPD2) axis, which reprograms cellular metabolism and can promote tumour proliferation and tumour metastasis.^91^ It has been found that ATG16L1‐deficient macrophages increase the expression of pro‐inflammatory factors IL‐1β and IL‐18 compared to autophagy gene‐intact mice, possibly because autophagy avoids the accumulation of intracellular DAMPs^92^ (Figure 3).

### 3.2 Ferroptosis mediates TIME reprogramming of macrophages

A mounting number of studies have reported that macrophages in the TME can undergo reception and integration of signals from the environment, communicate at the cell‐to‐cell level and successfully interconvert between different polarization states.^93^ Following this, macrophages of different polarization types remodel the tumour immune microenvironment from different perspectives. Iron‐dead tumour cells release oxidized lipids, eicosanoids (5‐HETE, 11‐HETE and 15‐HETE), prostaglandins (PEG) and DAMPs as signalling messengers interacting with other components in the TME.^94^ Intercellular horizontal communication may result in accumulation of large amounts of neutral lipids by DCs, leading to inhibition of their cross‐presentation function and impaired anti‐tumour immunity.^95^ Interestingly, PEG2 not only inhibits chemokines such as CCL5 to reduce DC infiltration TME but also has been shown to have an inhibitory effect on NK cell function.^96^ Tumour cell ferroptosis is followed by an effect on the polarization progression of TAMs. DAMPs can interact with pattern recognition receptors (PRRs) on TAMs to mediate macrophage differentiation in the M1 and M2 direction. The signals decoded by DAMPs after binding to PRR are mainly divided into ‘eat‐me’ and ‘save‐me’. The ‘eat‐me’ signals mainly synergize with anti‐tumour immunity, remove tumour cells and promote macrophage M1 polarization. Common ‘eat‐me’ signals include 1‐steaoryl‐2‐15‐HpETE‐sn‐glycero‐3‐phosphatidylethanolamine (SAPE‐OOH) and high mobility group box 1 (HMGB1).^97^, ^98^ HMGB1 binds to advanced glycosylation end product‐specific receptor (AGER) to release TNF‐α synergistically for anti‐tumour immunotherapy. SAPE‐OOH and toll‐like receptor 2 (TLR2) mediate macrophage phagocytosis of tumour cells to inhibit tumour progression.^97^ The ‘save‐me’ signal mainly initiates tissue repair, maintains the tumour immunosuppressive microenvironment (TIME) and converges macrophages towards M2 polarization.^99^ Pancreatic ductal adenocarcinoma (PDAC) tumour cells upon ferroptosis release KRASG12D in the form of exosomes that are accepted by AGER to promote macrophage M2 polarization while releasing arginine (ARG), IL‐10 and TGF‐β to construct and maintain TIME.^100^ In addition, the formation of 8‐hydroxy‐2′‐deoxyguanosine (8‐OHG) as a result of DNA damage due to oxidative damage can affect macrophage polarization through direct action on STING1.^101^ Notably, ferroptosis in macrophages itself is downregulated by TP53 diversity and hypoxia‐induced nuclear receptor coactivator 4 (NCOA4) resulting in a sluggish state for ferroptosis to occur.^31^, ^100^ Macrophages are involved in the processing of erythrocyte iron homeostasis, which may lead to suppression of autoimmune activity.^102^

**FIGURE 4:**
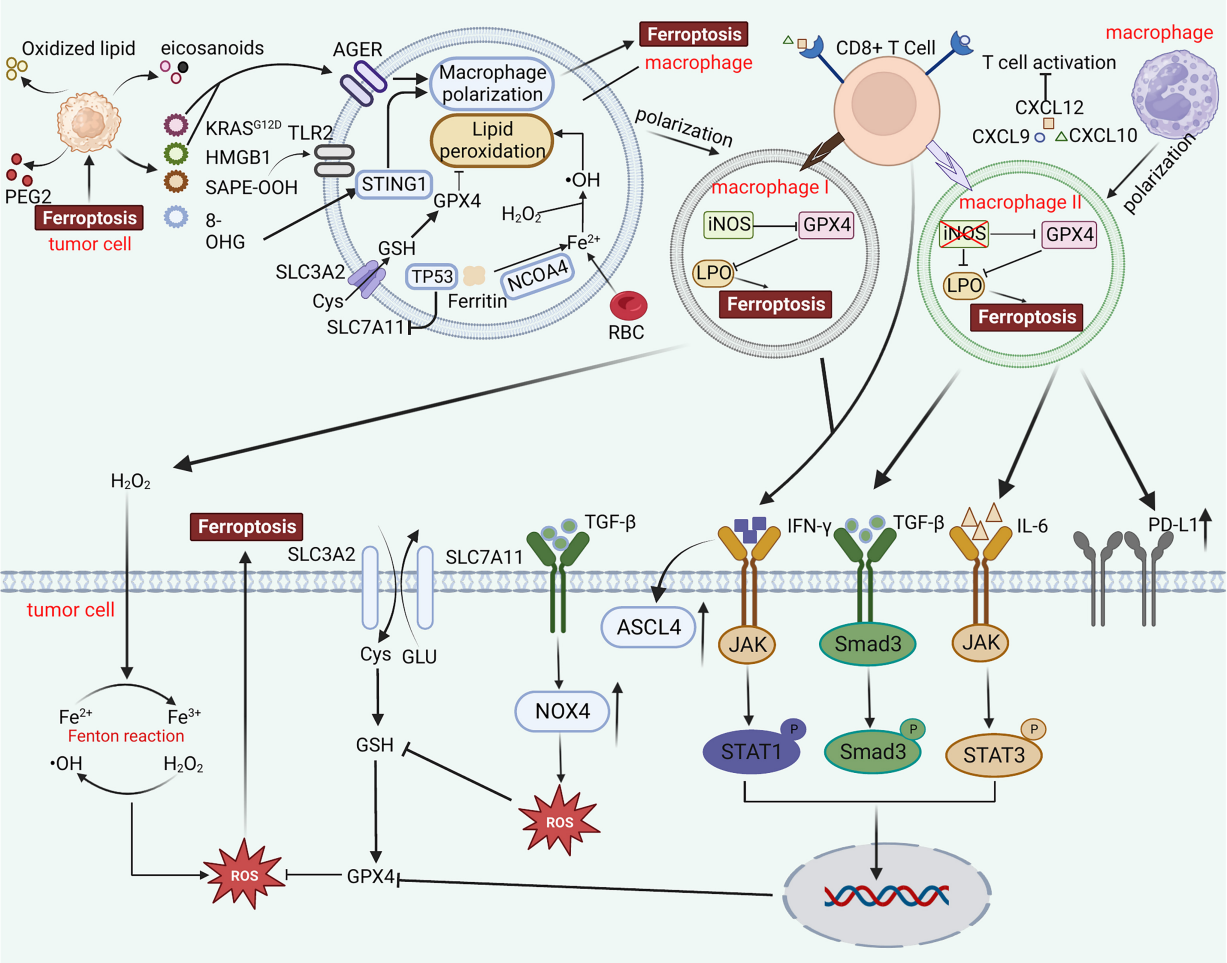
Ferroptosis mediates macrophage reprogramming of the tumour immune microenvironment. DAMPs released by ferroptosis of tumour cells will affect the polarization direction of macrophages in TME. In addition, the spontaneous ferroptosis of macrophages has a strong correlation with maintaining the iron balance of red blood cells, TP53 polymorphism and downregulation of NCOA4 expression. Polarized macrophages (M1 phenotype and M2 phenotype) in tumour microenvironment can play a role in direct contact with CD8+T cells. M1 phenotype can activate CTL to secrete IFN‐γ and inhibit the function of XC antioxidant system and produce a large number of peroxides to promote Fenton reaction and ROS accumulation in tumour cells, thus accelerate ferroptosis in tumour cells. M2 phenotype can directly contact T cells to inhibit the activation of CTL reduce the production of IFN‐γ and play an immunosuppressive role. Paradoxically, TGF‐β and IL‐6 secreted by M2 phenotype play an anti‐tumour role in XC antioxidant system by inhibiting the transcription of SLC7A11 and the function of GPX4, respectively.

M1 cells are more resistant to ferroptosis than M2 cells under natural conditions even in the absence of the GPX4 antioxidant system. This phenomenon arises because M1 cells are able to produce nitric oxide synthase (iNOS) and have more NO radicals (NO•) for antioxidant protection whereas M2 cells do not have this ability.^103^ TAMs crosstalk with tumour cells in three main ways: mediation by T cells, cytokines and direct activation.^104^, ^105^ M1 cells activate CD8+ cytotoxic T lymphocytes (CTL) driving them to release more interferon gamma (IFN‐γ) through cell contact‐dependent activation.^106^ IFN‐γ accomplishes its mission mainly through two mechanisms: inhibition of the xc‐system and enhancement of ACSL4 activity.^107^, ^108^ The former inhibits SLC7A11 transcription and assembly of the xc‐system by activating the Janus kinase/signal transducer and activator of transcription 1 (JAK/STAT1) pathway, which causes problems in the tumour antioxidant system.^109^ In the latter case, ACSL4 activity rises, promoting the accumulation of lipid peroxides and impaired resistance to tumour fine ferroptosis.^110^ It has been reported that during the interaction of M1 cells with TME, M1 cells release large amounts of peroxides during the respiratory burst, which directly promotes the Fenton reaction within the tumour cells and promotes the accumulation of ROS.^111^ It is well known that M2 cells are associated with the maintenance of TIME and one of the components of resistance to anti‐tumour therapy. M2 cells can secrete a variety of chemokines such as C‐X‐C motif chemokine ligand 9 (CXCL9), C‐X‐C motif chemoattractant cytokine ligand 10 (CXCL10) and C‐X‐C motif chemoattractant cytokine ligand 12 (CXCL12) to block CTL aggregation and activation.^112^ This is indeed not good news for the occurrence of ferroptosis in tumour cells. In addition to this, to help tumour cells resist ferroptosis, M2 cells promote the expression of PD‐L1 on the surface of tumour cells, which is a second strategy to rescue tumour cells.^113^ Tumour cells that highly express PD‐L1 bind to PD1 on the CTL to initiate programmed death of the CTL.^114^ This has been shown to be one of the reasons for the resistance to anti‐PD‐L1 therapies.^115^ Interestingly, although M2 is thought to be a weapon of tumour cells against ferroptosis, however, some of cytokines secreted by M2 have the effect of accelerating ferroptosis in tumour cells. For example, TGF‐β1 can activate NADPH oxidase 4 (NOX4), which allows NADPH to carry out electron transfer, causing accumulation of large amounts of ROS in tumour cells.^116^ Meanwhile, TGF‐β1 downregulates SLC7A11 transcription in the nucleus by affecting Smad3, the xc‐system antioxidant dysfunction, and the ferroptosis process is accelerated.^117^ The ability of IL‐6 to initiate ferroptosis is equally striking, which can disrupt intracellular iron homeostasis via JAK/STAT3 while using protein kinase (ERK) as a signalling amplifier inhibition of the antioxidant function of the xc‐system catalyses ferroptosis in tumour cells.^118^, ^119^ M2‐type macrophages exhibit tumour suppressive roles that are different from our previous perceptions, opening new horizons of tumour treatment^119^ (Figure 4).

### 3.3 Pyroptosis mediates TIME reprogramming of macrophages

Recently, several studies have pointed out that tumour cell pyroptosis triggers the entry of a large number of immune cells into the tumour microenvironment, which promotes the onset of tumour pyroptosis and establish positive feedback anti‐tumour immunity.^120^ Positive feedback anti‐tumour immunity induced by pyroptosis is so potent that only 15% of cancer cells need to undergo pyroptosis to achieve complete killing of the overall tumour.^121^ In fact, the release of the main effectors of pyroptosis (IL‐1β, IL‐18 and HMGB1) breaks down the ‘cold’ tumour properties and increases the infiltration of anti‐tumour immune cells in the TME, while at the same time decreasing the infiltration of suppressor immune cells.^122^ Therefore, pyroptosis can not only act directly on tumour cells but also promote the establishment of an anti‐tumour immune microenvironment, thus accomplishing the mission of tumour elimination in a dual dimension.

Inflammatory vesicle‐dependent IL‐1β and IL‐18 exert anti‐tumour effects or pro‐tumour effects mainly depending on the TME. Since it is known that IL‐1β and IL‐18 antagonism of tumour immune responses needs to be discussed depending on the type of cancer, it will not be discussed in detail here and has been summarized in an excellent review.^123^ IL‐1β and IL‐18 are initiating anti‐tumour immune indispensable core players. For example, IL‐1β is a key catalyst for DC maturation and can help accelerate the initiation of DC‐mediated adaptive immune response.^124^ Interestingly, ATP can bind to the P2X7 receptor on DCs to promote the secretion of IL‐1β by DCs, and the establishment of a positive cycle strengthens the anti‐tumour immune environment.^125^ Indeed, ATP can bind to another purinergic receptor, P2Y, to promote IL‐8 production and enhance neutrophil recruitment to help establish anti‐tumour immunity.^126^ IL‐18 has a role in promoting maturation as well as enhancing the function of NK cells and CD4+ T cells.^127^ In addition, HMGB1 released by pyroptotic cells is important for TME remodelling. HMGB1 seeks binding targets in the TME such as RAGE on tumour cells or RAGE on immune cells and TLR2/4 binding.^128^, ^129^ Interestingly, HMGB1 binding to different receptors produces different effects on the reconstitution of TIME. When HMGB1 binds to RAGE, it enhances extracellular regulatory protein kinase (ERK1/2) signalling by acting on Rac1 and Cdc42 to increase cell proliferation and migration.^130^ In addition, HMGB1 can also bind to TLR2/4 and regulate cytokine production by immune cells through activation of the transcription factors NF‐κB and AP‐1 simultaneously affecting the release of cytokines (IL‐6, TNFα, IL‐8).^128^

**FIGURE 5:**
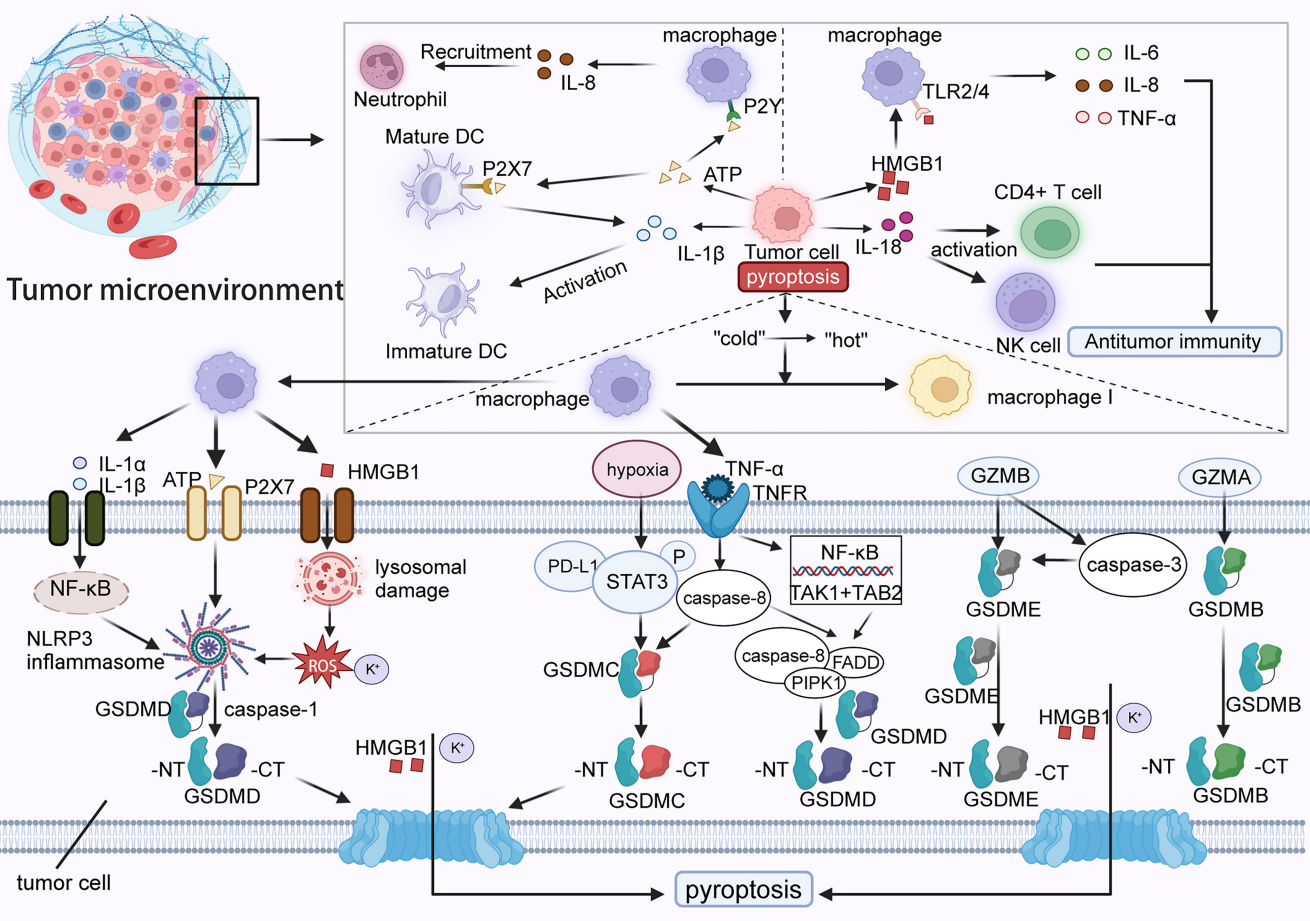
Pyroptosis mediates macrophage reprogramming of the tumour immune microenvironment. The effector of tumour cell pyroptosis has the ability to reshape tumour immune microenvironment. ATP initiates macrophages to secrete IL‐8 to recruit neutrophils for infiltration, and IL‐1β promotes antigen presentation mediated by DC cells to prepare for T‐cell infiltration. In addition, it can also promote the functional activation of NK cells and CD4+ T cells and chemotaxis of macrophages to polarize M1 phenotype. The secretion of macrophages can crosstalk with other tumour cells in tumour microenvironment, which promotes the progress of tumour cell pyroptosis. TNF‐α can bind to the receptor on the surface of tumour cells, activate the pyroptosis of tumour cells through two different pathways and enhance the occurrence of anti‐tumour immune response. In addition, IL‐1α, IL‐1β, ATP and HMGB1 can all complete the pyroptosis of tumour cells by stimulating inflammatory body NLRP3. It is worth noting that GZMB has higher cutting efficiency than GZMA, which is helpful to inhibit tumour progression.

The above discussed the ways and effects of tumour cell pyroptosis in reprogramming the tumour immune microenvironment, however, TAMs are important for promoting tumour cell pyroptosis. TAMs can affect tumour cell pyroptosis through multiple pathways, in which inflammatory vesicles, cytokines and granzymes are important players. TAMs are able to affect tumour cell pyroptosis by, respectively, affecting the relevant members of the classical and nonclassical pathways.^131^ Recent studies have pointed out that TAMs have an important role in the activation of inflammatory vesicles in tumour cells, which in turn helps tumour cell death and creates an anti‐tumour effect. First, activation of TAMs releases IL‐1α and IL‐1β to act on tumour cells, activating the intracellular NF‐κB pathway, which next exerts a pro‐coagulant effect by affecting NLRP3 inflammatory vesicles.^132^ Secondly, damaged or juxtaposed TAMs release DAMPs to bind tumour cell‐associated receptors, leading to lysosomal disruption, ROS release and K+ efflux in tumour cells, followed by activation of NLRP3 inflammatory vesicles.^133^ Similarly, TAMs can promote the release of ATP, and the rise in ATP concentration in the TME binds to tumour cell P2X7 receptors and opens cell surface K+ channels, which in turn promotes the activation of NLRP3 inflammatory vesicles and induces tumour cell pyroptosis.^134^ The above discussion demonstrates that TAMs can affect the activation of tumour cell inflammatory vesicles in the classical pathway through different mediators to induce tumour cell pyroptosis. Hypoxic properties in TME catalyse the binding of PD‐L1 and p‐STAT3 in the nucleus of tumour cells, leading to nuclear translocation and increasing GSDMC expression. Macrophage‐secreted TNF‐α binds to TNFR on the surface of tumour cells and induces caspase‐8 cleavage of GSDMC, reversing the apoptotic pathway into pyroptosis and contributing to anti‐tumour immunity.^135^ Interestingly, TNF‐α does not appear to be the only pathway mediating caspase‐8‐dependent mediation of tumour cell pyroptosis. It has been shown that TNF‐α binding to TNFR1 activates TAK1 to bind to TAK1‐binding protein 2 (TAB2) to induce tumour cell pyroptosis using the PIPK1, caspase‐8 and FADD protein complex as a tool.^136^ Granzyme B (GZMB), a serine protease, can be stored in a variety of immune cells (macrophages and T cells, among others). Interestingly, granzyme B in TME can target macrophages, which release large amounts of pro‐inflammatory factors, leading to a cytokine storm. GSDME has been reported to be suppressed in the expression of a variety of cancer cells, and low expression of GSDME was found to be associated with a better prognosis in breast cancer; thus, GSDME may be a tumour suppressor.^131^ In addition, cleavage of GSDME by GZMB promotes tumour cell death and enhances the establishment of anti‐tumour immune effects. The efficacy of the GZME‐GSDME axis relies mainly on the direct cleavage of GSDME by GZME; however, the cleavage of GSDME can also be accomplished by indirect activation of capase‐3.^120^ Similarly, Granzyme A cleaves GSDMB to promote tumour cell death in tumour cells with high expression of GSDMB, which has been shown to be highly expressed in cancer and associated with tumour metastasis. Notably, the presence of IFN‐γ in TME can promote the expression of GSDMB in tumour cells and the GZMA‐GSDMB axis efficacy. It is important to point out that there is a gap between the cleavage ability of GZMA and GZMB^137^, ^138^ (Figure 5).

### 3.4 Necroptosis mediates TIME reprogramming of macrophages

In 2016, Aes et al. demonstrated for the first time that necroptosis of cancer cells promotes DC cell maturation, CTL activation and IFN‐γ production, which illustrates the relevance of necroptosis as a form of immunogenic cell death concurrent with anti‐tumour immune activation.^139^ Interestingly, necroptosis‐associated effectors were shown to better alter the activity of immune cells in the TME to mediate the onset of anti‐tumour immunity compared to DAMPs released by necroptosis of cancer cells. The fact that supports this view is that DAMPs released by necroptosis of cancer cells alone are not sufficient for CTL cross‐initiation, but are required for cellular transcription through RIPK1 signalling and NF‐κb in dying cells.^140^ Similarly, NF‐κb signalling pathway, rather than MLKL, mediates necroptosis in fibroblasts in TME. However, a stronger anti‐tumour immune response will be induced.^141^ In addition, RIPK1 and RIPK3 are strongly correlated with high levels of tumour migration and metastasis in pancreatic cancer.^142^ Taken together, necroptosis‐associated effectors elicit anti‐tumour immunity differently in different cancers in terms of both effects and intensities and may be more dependent on the environment in which the cancer cells are located. Furthermore, RIPK1/3 was shown to be independent of necroptosis to mediate anti‐tumour effects and inflammatory effects in TME.^143^

Macrophages have been shown to remodel the immune microenvironment in TME through necroptosis‐associated effectors (RIPK1/RIPK3) and towards immunosuppression. RIPK1/RIPK3 expression levels have been reported to be higher in PDA than in peripheral pancreatic tissues. Furthermore, the knockdown or inhibition of RIPK1 has the ability to avoid krasg12d‐promoting tumour progression.^144^ In constructed mouse models, RIPK1/RIPK3 has the ability to enhance the expression of sin3A‐associated protein 130 (SAP130), CXCL1 and CXCL5, leading to the phenomenon that macrophages polarize towards M2 direction while promoting immunosuppression and tumour metastasis mediated by M2‐type cells.^144^, ^145^ Furthermore, M2‐type cells polarized to immunosuppression were shown to have elevated expression of RIPK1 in a PDA model, which can restart adaptive immune responses and inhibit tumour development by inducing immune reprogramming of immunosuppressed M2‐type cells using gsk547, an inhibitor of stat1.^146^ Notably, RIPK1 inhibition leads to the fusion of cytotoxic T cells with T helper cells into a Th1/Th17 cell phenotype, allowing anti‐tumour immunity to be activated to inhibit tumour growth.^147^ Interestingly, the pro‐tumorigenic effect of RIPK1 does not synergize with RIPK3 but exists independently. It is not difficult to see that targeting RIPK1/RIPK3 in macrophages has the potential to be a potent target to restrain tumour progression. Recent studies have reported that treatment of a mouse model of spontaneous intestinal tumours with gsk872 (RIPK3 kinase inhibitor) significantly revealed a significant reduction in the number of intermediate MDSCs (I‐MDSCs) and macrophages. The primary mechanism is that IL‐17 production of I‐MDSCs are mediated by RIPK3 and thus mediate the development of intestinal tumour.^148^

Macrophages play a role in the regulation of the immune microenvironment in the TME through their own necroptosis‐related effectors. The release of DAMPs or cytokines by necroptotic apoptosis of tumour cells and the phagocytosis of cancer cells by macrophages still have a remodelling role for the TME through their interaction.^149^ How classical DAMPs (HMGB1, ATP) act on macrophages to remodel TME has been described in detail elsewhere in this paper. In necroptosis, we focus on the remodelling effects brought about by cytokines and phagocytosis effects. CXCL1 is one of the cytokines secreted by necroptotic cancer cells, which can maintain an immunosuppressive microenvironment in the TME by allowing a high degree of MDSC and TAM passage yet limiting the infiltration of hyper‐immunogenic T cells and B cells.^144^ In addition, a large number of macrophages recruited by chemokines or ATP can secrete cytokines, such as TNF, into the TME, which can bind to TNFR1 on cancer cells with normal state and initiate a new necroptosis, establishing a positive feedback effect and inhibiting cancer progression.^150^ Similarly, it has been demonstrated at the transcriptomic level that TNF‐induced necroptosis interacts with pro‐inflammatory NF‐κB pathways, increasing the expression levels of pro‐inflammatory factors and establishing anti‐tumour immunity.^151^ When APC phagocytoses cancer cells that undergoing necroptosis and will directly promote the maturation of DCs to effectively present tumour antigens to CD8+ T cells, enhancing immune response and IFN‐γ production. It is important to note that necroptosis of cancer cells has a tendency to preferentially recruit macrophages, which may also be an indication of vertebrate conservatism.^152^ Interestingly, for the recruitment requests of necrotic apoptotic cancer cells, it is likely that the ones sending the resume will be immunosuppressive cells such as M2‐type macrophages, Treg and MDSCs. Their arrival establishes and maintains the generation of an immunosuppressive microenvironment that promotes tumour angiogenesis, metastasis and infiltration.^153^ A rapid increase in the content of reactive nitrogen intermediates (RNI) and ROS in the TME due to necroptosis of cancer cells also participates in the pro‐tumorigenic process.^144^

Above we described that necroptosis produces a dual effect on TME. However, it has been reported that necroptosis‐mediated ICD is more likely to reprogram the TME towards immunosuppressive effects rather than immune support. For example, regardless of which cytokine initiates necroptosis, the ability of necroptotic apoptotic cells for long‐term support of the CTL response still needs to be questioned, even to the extent of releasing cytokines capable of blocking CTL cross‐initiation and avoiding CTL infiltration into cancerous tissues.^154^ In addition, the TNF‐driven necroptosis pathway would inhibit the release of large amounts of pro‐inflammatory factors through RIPK3‐dependent features, remodelling TIME towards immunosuppression.^155^ In the previous discourse, we were more inclined to discuss the evidence for the generation of an immunosuppressive microenvironment supported by necroptosis‐mediated unidirectional immunity, which would become more complex if discussed in the context of tumour TME. For example, IL‐1α a cytokine released by necrotic tumour cells can promote stromal cell proliferation, and stromal endogenous factors secreted by stromal cells counteract the tumour to promote angiogenesis, metastasis and infiltration.^156^

### 3.5 Other non‐apoptotic regulated cell death mediates TIME reprogramming of macrophages

The study reported that microarray analysis of PARP‐1 gene expression in 8000 samples indicated that PARP‐1 expression was higher in several types of cancers than that in normal tissues.^157^ For example, inhibition of PARP‐1 expression in epithelial cancers reduces tumour‐promoting proteins expressed by the NF‐κB pathway and reduces epithelial tumorigenesis.^158^ Thus, the process of PAR in carcinogenesis can be referenced in two dimensions. First, due to the DNA repair role of the PAR family, it can promptly repair DNA damage in cancer cells due to chemotherapy and radiotherapy and maintain the morphology and function of cells. Secondly, the overactivation of PARP‐1 activity contributes to the development of parthanatos and inhibits the proliferation of tumour cells.^159^ So far, the specific molecular characterization of the parthanatos cell death pathway in MΦs remains to be explored. However, there is a clear consensus that with the help of PARP1, parthanatos can release DAMP proteins, among which HMGB1 is the most exemplary representative. HMGB1 is dependent on the transfer of PARP1 from the nucleus to the cytoplasm of the cell, waiting to be released upon rupture of the plasma membrane, which demonstrates the ability of MΦs to manipulate the onset of inflammatory responses. HMGB1 is important for the activation of innate immune cells and cytokine production in TIME, and in turn, parthanatos functions to regulate macrophage reprogramming of TIME.^160^

The process of entotic cell death directing tumours is two‐dimensional. Although it leads to an aneuploid shift in phagocytes (tumour cells) while providing them with food for growth, it also promotes the killing of cancer cells by the remaining cells in TIME.^161^ Notably, the process by which immunotoxic cells actively enter tumour cells in TIME and die therein is termed emperitosis. Emperitosis refers to the structure of heterogeneous cell in cell. The mechanism of emperitosis of immunotoxic cells is that after entering the tumour cells they are submerged in the tumour cell vesicles and further GzmB degranulation in the vesicles is re‐uptaken by the immunotoxic cells, triggering apoptotic signalling pathways within the immunotoxic cells not lysosomal‐mediated death.^162^, ^163^ It is conceivable that macrophages in TIME have the same property of secreting GzmB, and the occurrence of emperitosis greatly inhibits TIME viability, which in turn accomplishes tumour immune escape.

It has been shown that elevated copper levels in tumour cells are highly correlated with tumour revascularization and support tumour progression. In addition, vascular endothelial growth factor (VEGF) in hepatocellular carcinoma can convene immunosuppressive cells (M2‐type macrophages, MDSCs, Tregs) in TME to maintain negative TIME.^164^ Interestingly, copper content in TME was positively correlated with NK cells, CD8+ T cells and neutrophil infiltration, and conversely, negatively correlated with M2 macrophages, and MDSCs infiltration, which implies that induction of tumour cell cuproptosis contributes to tumour elimination.^165^

NETosis is found in a variety of ‘cold’ tumours and may be associated with negative TME construction. In ovarian cancer, a typical ‘cold’ tumour, NETosis induced by neutrophils, a member of the TME, facilitates the construction of pre‐metastatic ecological niches in the leptomeninges, which contributes to immune escape of tumour cells.^166^ Furthermore, it was found that in Nets isolated from colorectal cancer patients with liver metastases, NETosis can induce CD4 and CD8 T‐cell exhaustion in the tumour microenvironment.^167^ Similarly, NETosis is induced by G‐CSF, IL‐8 and chemokines (CXCR1, CXCR2) in the TME, which can physically segregate cancer cells from immunotoxic cells through the encapsulation of nets.^168^ Finally, NETosis not only leads to negative TIME but is also highly correlated with tumour cell proliferation, metastasis and drug resistance, sustaining tumour survival in multiple dimensions.

## 4 TARGETED NON‐APOPTOTIC RCD THERAPY COMBINED WITH ICIs

### 4.1 Recent advances in anti‐tumour drugs targeting autophagy, ferroptosis, pyroptosis and necroptosis

Targeting autophagy, ferroptosis, pyroptosis and necrosis drugs can affect tumour progression by promoting (inhibiting) the development of non‐apoptotic RCD in cancer cells. The following is a review of some of the drugs that have been used in the clinic to target non‐apoptotic RCD. Chloroquine derivatives, mainly categorized as chloroquine (CQ) and hydroxychloroquine (HCQ), are currently the only clinically approved autophagy inhibitors, which inhibit autophagy mainly by inhibiting the degradation function of lysosomes on autophagosomes.^169^ Unlike autophagy inhibitors that act at the lysosomal site, autophagy inhibitors that target P13K can inhibit autophagy at an early stage, and common P13K inhibitors include 3‐methyladenine (3‐MA), LY294002.^170^, ^171^ In view of the dual manifestation of autophagy in tumour growth, the use of autophagy activators in cancer should not be ignored. Lipocalin (ADIPOQ) and 2‐aminonicotinonitrile compounds (w09), both autophagy activators, were reported to drive autophagy genesis in cancer cells through the STK11/LKB1‐AMPK‐ULK1 and EGFR‐RAS‐RAF1‐MAP2K‐MAPK1/3 pathways, respectively, and inhibit the progression of breast cancer and gastric cancer, respectively.^172^, ^173^

Based on the molecular mechanisms of ferroptosis, there 'are two main drug‐targeting approaches. The first is to restrict the work of the xc‐system so that cystine transport is impaired, increasing the aggregation of ROS in cancer cells and inducing ferroptosis. Sorafenib, a drug that classically disrupts the work of the xc‐system, deplete intracellular GSH mainly through inhibition of cystine‐glutamate exchange, thus inhibits the activity of the xc‐system and increases the aggregation of ROS in cancer cells. It has been mainly used in the treatment of renal cell carcinoma, HCC and thyroid cancer, among others.^174^ However, many cancers become resistant to Sorafenib, mainly due to other alternative antioxidant systems (thioredoxin antioxidant is not dependent on the GSH pathway) and so on. In response to this phenomenon, ovarian can directly target GPX4 to disable antioxidant systems with compensatory effects, and significantly inhibited tumour progression.^175^ The second drug‐targeting approaches are by disrupting the iron homeostasis in cancer cells, thus increase free iron in cancer cells and induce ferroptosis. Artemisinin compounds and cisplatin can induce ferroptosis in cancer cells by modifying the ratio of iron‐responsive proteins to iron‐regulatory proteins in cancer cells and adjusting the intracellular free iron content, which is extremely helpful for targeted therapy of lung cancer.^176^, ^177^

The main approach among anti‐tumour drugs targeting pyroptosis is to find drugs that can efficiently cleave the Gasdermin family to promote cancer cell pyroptosis. α‐NETA has been reported to promote epithelial ovarian cancer (EOC) cell pyroptosis via the caspase‐4/GSDMD pathway.^178^ The PLK1 inhibitor, BI2536 and the alkylating agent, cisplatin, promoted the development of tubular squamous cell carcinoma (ESCC) into pyroptosis and inhibited tumour progression by augmenting the caspase‐3/GSDME pathway.^179^ In addition, miltirone, an active compound from the traditional Chinese medicine Salvia miltiorrhiza, triggered caspase‐3/GSDME to drive the realization of HCC pyroptosis through ROS accumulation. It was found that the main mechanism of miltirone is to activate the Bax pathway by triggering ROS to further activate caspase‐3, and the activated caspase‐3 will cleave GSDME to complete the core step of HCC scorched death.^180^ Polymyxin VI (PPVI) uses the ROS/NF‐κB/NLRP3/GSDMD signalling axis to awaken caspase‐1 to complete the cleavage mission of GSDMD to promote pyroptosis in NSCLC cancer cells, demonstrating the potential of PPVI for inhibiting NSCLC progression.^46^ It is worth mentioning that metformin seems to accomplish the mission of tumour cell pyroptosis through the MiR‐497/PELP1/GSDMD signalling axis. However, it is not clear which protease is commanded to cleave GSDMD, and subsequent studies are necessary in view of the wide range of applications and safety features.^181^ Necroptosis in apoptogenesis critically relies on RIPK1/RIPK3/MLKL to mediate its occurrence; however, these molecules are mainly controlled by ROS and caspases. Biological selenium nanoparticles (SeNPs) can significantly stimulate necroptosis in prostate adenocarcinoma cells. The main mechanism is that after treatment with SeNPs, elevated ROS accumulation in cancer cells activates TNF and interferon regulatory factor 1 (IRF1), which ultimately leads to an increase in RIP1 protein expression inducing necroptosis.^182^ Shikonin is the first drug found to act in the RIPK1/RIPK3‐dependent necroptosis pathway and promotes tumour necroptosis.^183^

### 4.2 ICIs target autophagy, ferroptosis, pyroptosis and necroptosis

Immune checkpoint inhibitors (ICIs) are used to indirectly achieve tumour growth inhibition by interacting with immune checkpoints on the surface of tumour cells and awakening the immune system's ability to re‐recognize the tumour. However, ICIs are not a panacea, and lack of response beyond more than 2/3 of the patient population is a major limitation.^184^ Resistance arises mainly characterized by lack of CTL cell infiltration in TME and infiltration of immunosuppressive cells (MDSCs, TAM, Treg) forming an immunosuppressive microenvironment, making the efficacy not as good as we expected.^185^ Therefore, an attempt was made to propose the combination of ICIs and ICD‐inducing (inhibitory) agents in anticipation of producing good results. As discussed in the autophagy section of this paper, autophagy of immune cells may play an antagonistic role in anti‐tumour responses, and inhibiting autophagy of immune cells is an ideal approach. In addition, in breast cancer models, CQ‐induced systemic autophagy inhibition did not affect T‐cell activity, suggesting that the immune system is tolerant to autophagy. For example, in a mouse pancreatic cancer model, treatment with CQ in combination with dual ICIs yielded promising results, with CQ inhibiting macrophage degradation of MHC‐I molecules, enhancing antigen presentation, and assisting in the anti‐tumour response.^74^ In fact, in melanoma models, as the VPS34 kinase inhibitors SB02024 or SAR405 induced a rise in CCL5, CXCL10 and IFN‐γ in the TME along with an increase in the extent of NK and T‐cell infiltration, the inhibition of melanoma was greatly enhanced. This phenomenon was also validated in a colorectal cancer model, and it is noteworthy that VPS34 inhibitors reversed anti‐PD1 or anti‐PDL1 resistance.^186^ It has been reported that high doses of IL‐2 can cause systemic autophagy syndrome with increased levels of IFN‐γ, IL‐6 and IL‐18 expression.^187^ Therefore, the combination of CQ and high‐dose IL‐2 elicits potent anti‐tumour immunity in a way that can avoid toxicity, which has yielded promising results in renal cell carcinoma and melanoma.^188^

Ferroptosis plays a huge role in disrupting the immunosuppressive microenvironment formed by immunosuppressive cells and direct the immune environment of TME to an anti‐tumour immune microenvironment.^189^ NC06 acts on ASAH2 to disrupt the P53 pathway in MDSCs, further inducing the ferroptosis of MDSCs in TME to solve the problem due to the accumulation of MDSCs resulting in immunosuppressive properties.^190^ Interestingly, cholesterol aids tumour immune escape by forcing T cells to undergo ferroptosis. Cholesterol promotes T‐cell expression of CD36 to enhance uptake for fatty acids, leading to ferroptosis of T cells.^191^ Therefore, blocking CD36 on the surface of T cells in combination with anti‐PD‐1 immunotherapy can achieve good therapeutic results. It is worth mentioning that ZVI‐NP can convert immunologically heterogeneous M2‐type cells into M1 anti‐tumour‐type cells at the same time can reduce the level of Treg in the TME, in addition, blocking the expression of PD‐1 and CTLA4 on the surface of T cells avoids binding to the tumour cell surface receptor blocking the immune response.^192^ Because cyst(e)inase can promote ferroptosis by depleting intracellular cystine, the combination of cyst(e)inase or GPX4 inhibitor and PD‐L1 antibody can well inhibit tumour progression.^193^ Pyroptosis induces the release of large amounts of inflammatory cytokines and DAMPs helping to establish anti‐tumour immunity.

It has been reported that one of the strategies to address ‘cold’ tumours is to intervene with ICIs under conditions of targeted tumour cachexia, as neither ICIs nor targeted tumour cachexia alone are effective.^121^ Caspase‐3/GSDME pathway is more common in targeted therapy for cachexia, and the combination of BRAF inhibitors and MEK inhibitors (BRAFi + MEKi) has been approved by the FDA for the treatment of melanoma with promising results.^194^ More commonly, the combination of BRAF inhibitors and MEK inhibitors (BRAFi + MEKi) has been approved by the FDA for the treatment of melanoma achieving favourable results.^194^ The main mechanism of action is that (BRAFi + MEKi) promotes the cleavage of GSDME while inducing pyroptosis and release of DAMPs to activate the activity of immune cells in the TME to cause anti‐tumour immunity. CD39/CD73 can degrade ATP released from pyroptosis cells firstly to AMP by CD39, and then, CD73 can continue to degrade the AMP known as adenosine, so CD39/CD73 can play a role by destroying ATP. CD73 exerts anti‐tumour effects by destroying ATP.^195^ Anti‐PD‐L1 efficacy is facilitated by the use of CD39/ CD73‐targeted drugs that synergistically promote tumour cell pyroptosis.^195^ Worryingly, the induction of pyroptosis does not always contribute to immunotherapy. One study demonstrated that CAR‐T cells mediate the GZMB/GSDME/CASP3 signalling axis to accomplish the pyroptosis of tumour cells. However, the released inflammatory factors activate caspase‐1 in macrophages to cleave intracellular GSDMD, leading to a dangerous side effect cytokine release syndrome (CRS).^196^ Necrosis‐inducing agents in combination with ICIs therapy are also effective. SMAC mimics modulate inhibitors of apoptosis proteins (IAPs) to induce cell necrosis. In a melanoma model, the SMAC mimetic birinapant increased the killing effect of T cells on tumour cells and enhanced the effect of ICIs by directly elevating the activity of immune cells through the NF‐κB signalling pathway.^197^ In addition, anti‐PD‐1 efficacy was improved in a mouse tumour model by using Smac mimics to mediate RIPK1‐dependent cell death to activate the activity of NK cells and T cells in the TME.^198^ (Table 1).

**TABLE 1:** Targeted non‐apoptotic RCD therapy combined with ICIs.

Drug name	Current progress	Effects on tumour cell death	Effects on immune response	Cancer type	References
HCQ/CQ	Clinical trial evaluation	Autophagy inhibition	Enhanced anti‐tumour immune response	Glioblastoma/NSCLC/breast cancer	[169]
3‐methyladenine/LY294002	In vitro study	Autophagy inhibition	Enhanced anti‐tumour immune response	Oesophageal Squamous Cell Carcinoma/ Glioblastoma	[170, 171]
SB02024/SAR405	In vitro study	Autophagy inhibition	Enhanced anti‐tumour immune response	Melanoma/Colorectal cancer	[186]
w09	In vitro study	Autophagy induction	Enhanced anti‐tumour immune response	Gastric cancer	[172]
ADIPOQ	In vitro study	Autophagy induction	Enhanced anti‐tumour immune response	Breast cancer	[173]
Sorafenib	Clinical trial evaluation	Ferroptosis induction	Enhanced anti‐tumour immune response	Hepatocellular carcinoma	[174]
Artemisinin compounds /cisplatin	In vitro study	Ferroptosis induction	Enhanced anti‐tumour immune response	\	[176, 177]
NC06	In vitro study	Ferroptosis induction	Enhanced anti‐tumour immune response	\	[190]
Cyst(e)inase	In vitro study	Ferroptosis induction	Enhanced anti‐tumour immune response	Prostate carcinoma	[193]
α‐NETA	In vitro study	Pyroptosis induction	Enhanced anti‐tumour immune response	Ovarian cancer	[178]
Miltirone	In vitro study	Pyroptosis induction	Enhanced anti‐tumour immune response	Hepatocellular carcinoma	[180]
Polymyxin VI	In vitro study	Pyroptosis induction	Enhanced anti‐tumour immune response	NSCLC	[46]
BRAFi + MEKi	Clinical trial evaluation	Pyroptosis induction	Enhanced anti‐tumour immune response	Melanoma	[194]
Selenium nanoparticles	In vitro study	Necroptosis induction	Enhanced anti‐tumour immune response	\	[182]
Shikonin	In vitro study	Necroptosis induction	Enhanced anti‐tumour immune response	Breast cancer	[183]

## 5 CONCLUSIONS AND PERSPECTIVES

Collating the latest clinical and experimental findings, this paper reviews how non‐apoptotic RCD mediates crosstalk between tumour cells and macrophages at the cellular level, further reprogramming the tumour immune microenvironment to impact tumour growth. In addition, the combination of directly targeting non‐apoptotic RCD and ICIs can effectively avoid the phenomenon that ICIs are effective only in part of the population. We focused on how the non‐apoptotic RCD of tumour cells and macrophages themselves reprogrammed the tumour immune microenvironment as well as further enhanced the effect of non‐apoptotic RCD of other tumour cells in the TME on tumour growth. Macrophage antigen presentation, cytokine secretion and polarization are altered under the mediation of non‐apoptotic RCD, further affecting both innate and adaptive immune responses.

In different tumour types, the role played by non‐apoptotic RCD switches back and forth between antagonizing and coordinating tumour growth, which leads us to be unable to determine the specific role of non‐apoptotic RCD. In addition, in this paper we only discussed the reprogramming of the tumour immune microenvironment by non‐apoptotic RCD crosstalk between macrophages and tumour cells. Although these two cells are the most abundant cells in the major cell populations and immune cell populations in the TME, respectively, however, other immune cells or stromal cells in the TME also undergo non‐apoptotic RCD exerting an influence on the immune response, and it is important to establish a three‐dimensional network of cellular communication that includes the stromal cells, tumour cells and all the immune cells in the TME. In the end, although direct targeting of non‐apoptotic RCD in combination with ICIs has yielded good results, this approach does not necessarily play a good role in tumour patients. The reason is that non‐apoptotic RCD can still occur in cell populations other than tumour cells in the TME, such as the reprogramming effect of non‐apoptotic RCD in macrophages on TIME. Therefore, it is urgent to try to develop non‐apoptotic RCD that can specifically target tumour cells.

## AUTHOR CONTRIBUTIONS

**Chengpeng Sun:** Data curation (equal); formal analysis (equal); investigation (equal). **Jianhao Zhan:** Data curation (equal); formal analysis (equal); investigation (equal); methodology (equal). **Yao Li:** Data curation (equal); methodology (equal). **Chulin Zhou:** Data curation (equal); formal analysis (equal); investigation (equal); methodology (equal). **Shuo Huang:** Data curation (equal); methodology (equal); project administration (equal). **Xingen Zhu:** Conceptualization (equal); data curation (equal); formal analysis (equal); funding acquisition (equal); investigation (equal). **Kai Huang:** Data curation (equal); formal analysis (equal); investigation (equal); methodology (equal); project administration (equal).

## FUNDING INFORMATION

National Natural Science Foundation of China (grant no. 82172989, 82273068 and 82260524), Key Research and Development projects in Jiangxi (grant no. 20212BBG73021), Jiangxi Training Program for academic and technical leaders of major disciplines—Young talents program (grant no. 20212BCJ23023), Key project of Science and Technology Innovation of Health Commission (grant no. 2023ZD003), Jiangxi Provincial Natural Science Foundation (grant no. 20232BAB206101), Jiangxi Province Department of Education Science and technology research project, China (grant no. GJJ210177), Project Fund of Jiangxi Provincial Health Commission (20204343, 202130873, 202210044) and The Second Affiliated Hospital of Nanchang University, National Natural Science Foundation incubation project (2021YNFY12010).

## CONFLICT OF INTEREST STATEMENT

The authors declare that the research was conducted in the absence of any commercial or financial relationships that could be construed as a potential conflict of interest.

## Data Availability

Data sharing not applicable to this article as no datasets were generated or analysed during the current study.
